# Complex bilateral coronary artery fistulas with multiple drainage sites: a multimodality imaging case report

**DOI:** 10.1093/ehjcr/ytag683

**Published:** 2026-09-24

**Authors:** Dušan Ružičić, Milenko Čanković, Nikola Komazec, Višeslav Popadić

**Affiliations:** Department of Invasive Cardiology, Health Center Valjevo, Sinđeliceva 62, 14000 Valjevo, Serbia; Faculty of Health Sciences, Singidunum University, Železnička 5, 14000 Valjevo, Serbia; Department of Cardiology, Institute of Cardiovascular Diseases of Vojvodina, Dr. Goldmana 4, 21204 Sremska Kamenica, Serbia; Faculty of Medicine, University of Novi Sad, Hajduk Veljkova 3, 21000 Novi Sad, Serbia; Department of Cardiology, Institute of Cardiovascular Diseases of Vojvodina, Dr. Goldmana 4, 21204 Sremska Kamenica, Serbia; Cardiology Clinic, Clinical Center ‘Bežanijska Kosa’, Dr Žorža Matea bb, 11080 Belgrade, Serbia

**Keywords:** Coronary artery fistula, Case report, Multimodality imaging, Coronary angiography, Computed tomography coronary angiography, Cardiac magnetic resonance

## Abstract

**Background:**

Coronary artery fistulas (CAFs) are rare coronary anomalies, most often detected incidentally. Although frequently asymptomatic, complex fistulas may lead to myocardial ischaemia, heart failure, or other complications, and optimal management remains controversial.

**Case summary:**

A 68-year-old patient presented with exertional dyspnoea and dizziness. Coronary angiography revealed intermediate left anterior descending artery stenosis with negative fractional flow reserve and a large CAF. Multislice computed tomography demonstrated complex bilateral CAFs originating from both coronary arteries, with drainage into the superior vena cava, right atrium, and descending aorta. Cardiac magnetic resonance imaging showed preserved myocardial tissue characteristics without ischaemia or fibrosis. Following Heart Team discussion, conservative management with optimal medical therapy was chosen. At 2-year follow-up, the patient remained asymptomatic without adverse events.

**Discussion:**

This case illustrates an exceptionally rare presentation of bilateral CAFs with multiple atypical drainage sites. It highlights the value of multimodality imaging for comprehensive anatomical and functional assessment and supports individualized, Heart Team–guided management in the absence of clear guideline recommendations.

Learning points

- Coronary artery fistulas (CAFs) may present with complex anatomy and atypical drainage sites involving both coronary arteries.
- Multimodality imaging is essential for anatomical and functional assessment of CAFs.
- In the absence of guideline-directed recommendations, management of CAFs should be individualized and guided by Heart Team decision-making.

## Introduction

Coronary artery fistula (CAF) is a rare congenital or acquired anomaly characterized by an abnormal communication between a coronary artery and a cardiac chamber or a great vessel. Although CAFs are often detected incidentally during coronary angiography or computed tomography (CT), most patients remain asymptomatic. However, in some cases, CAFs may lead to myocardial ischaemia, heart failure, pulmonary hypertension, arrhythmias, or sudden cardiac death. Due to the low prevalence of CAF and the lack of prospective studies, optimal management strategies remain controversial and are largely individualized.^1,2^

## Summary figure

**Figure:**
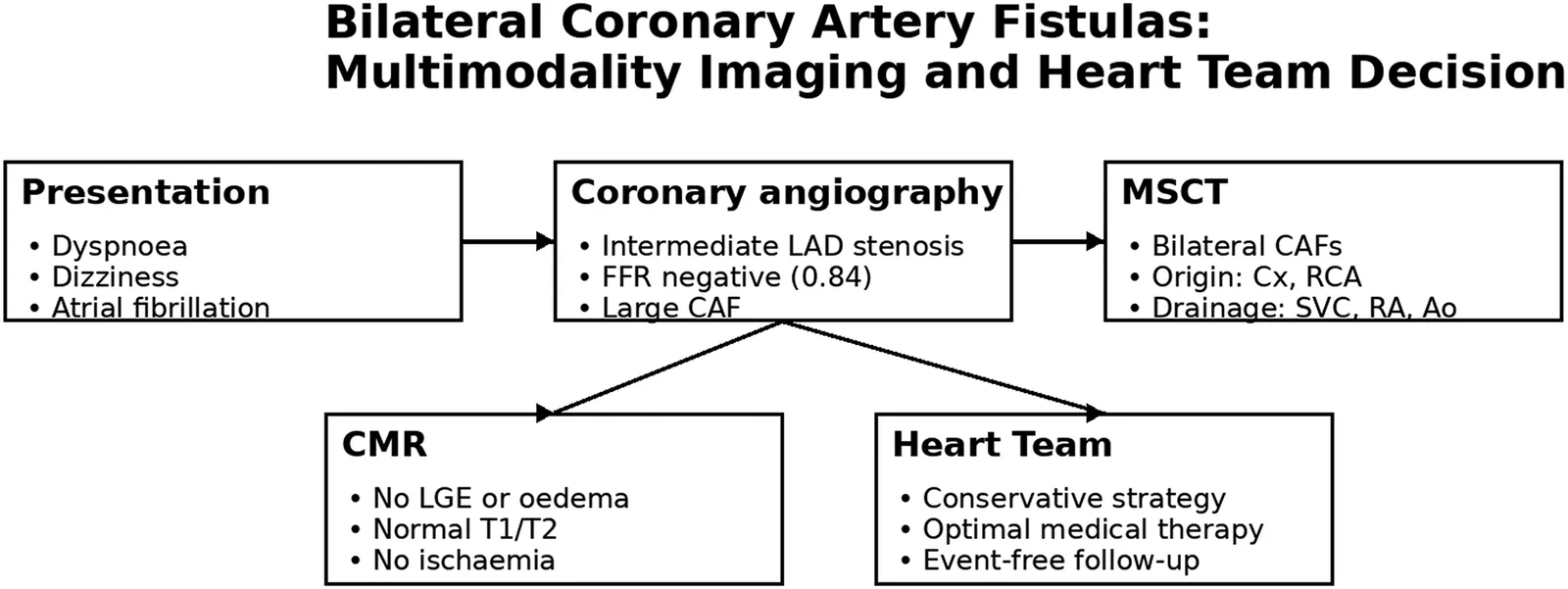


## Case presentation

**Figure 1:**
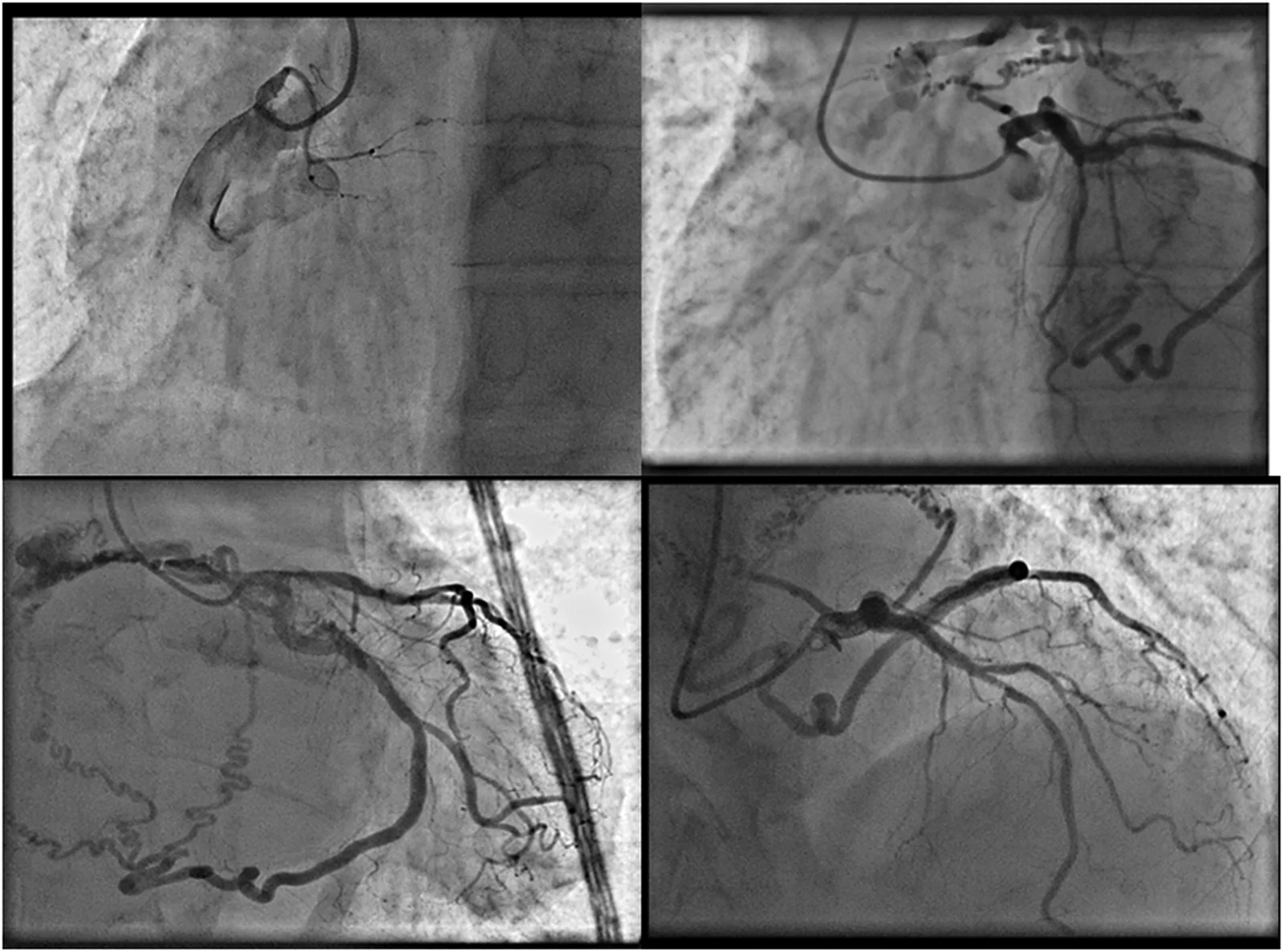
Coronary angiography demonstrating complex coronary artery fistulas. (*A*) Right coronary artery angiography showing contrast filling of a coronary artery fistula. (*B*) Left coronary artery angiography in spider view demonstrating the origin of the fistula from the circumflex artery. (*C*) Caudal projection of the left coronary artery illustrating the course of the fistula. (*D*) Cranial projection of the left coronary artery demonstrating late contrast opacification and drainage of the fistulous connection.

A 68-year-old patient with a history of arterial hypertension was admitted due to progressive exertional dyspnoea and dizziness. Physical examination was unremarkable. Electrocardiography showed atrial fibrillation without ischaemic changes. Transthoracic echocardiography demonstrated preserved left ventricular systolic function with an ejection fraction of 56% and no structural abnormalities. Stress echocardiography was positive for inducible ischaemia with hypokinesia of the basal segment of the inferior wall and the apical part of the septum, and the patient was referred for invasive coronary angiography. An intermediate stenosis of the left anterior descending artery was identified; however, fractional flow reserve (FFR) measurement was negative (FFR = 0.84). In addition, a large CAF was visualized (*Figure 1*).

**Figure 2:**
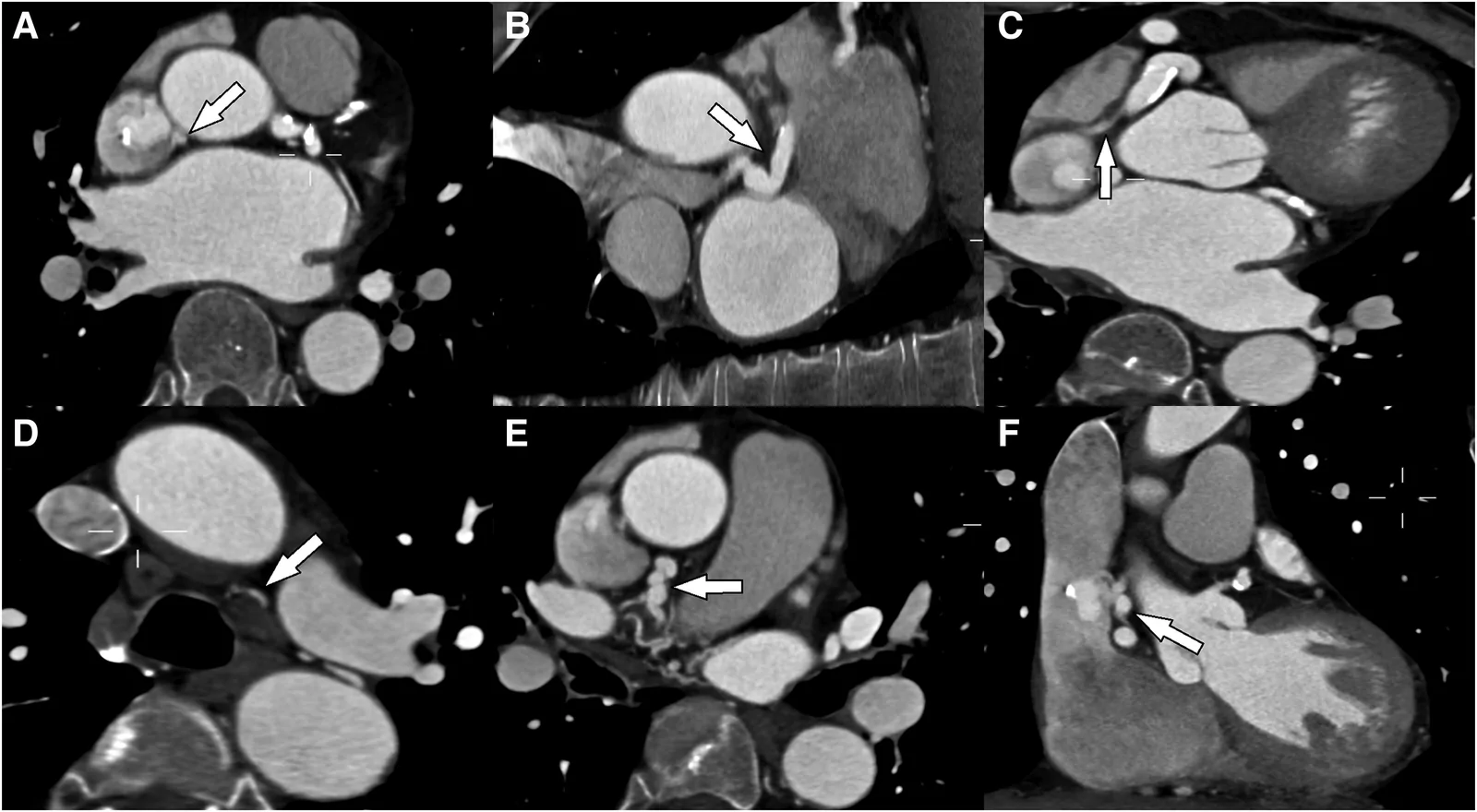
Computed tomography coronary angiography demonstrating complex coronary artery fistulas. Multislice computed tomography coronary angiography illustrating the complex anatomy and course of coronary artery fistulas. Arrows indicate the course of the fistulous communications and their drainage sites. (*A*, *B*) Axial images demonstrating the origin of the fistulous connection from the proximal circumflex coronary artery and its close relationship to adjacent cardiac structures. (*C*, *D*) Oblique and axial reconstructions showing the retroaortic course of the fistula with bifurcation and extension towards the venous system. (*E*) Axial image demonstrating fistulous drainage into the superior vena cava. (*F*) Multiplanar reconstruction illustrating the distal course and termination of the fistulous connection.

**Figure 3:**
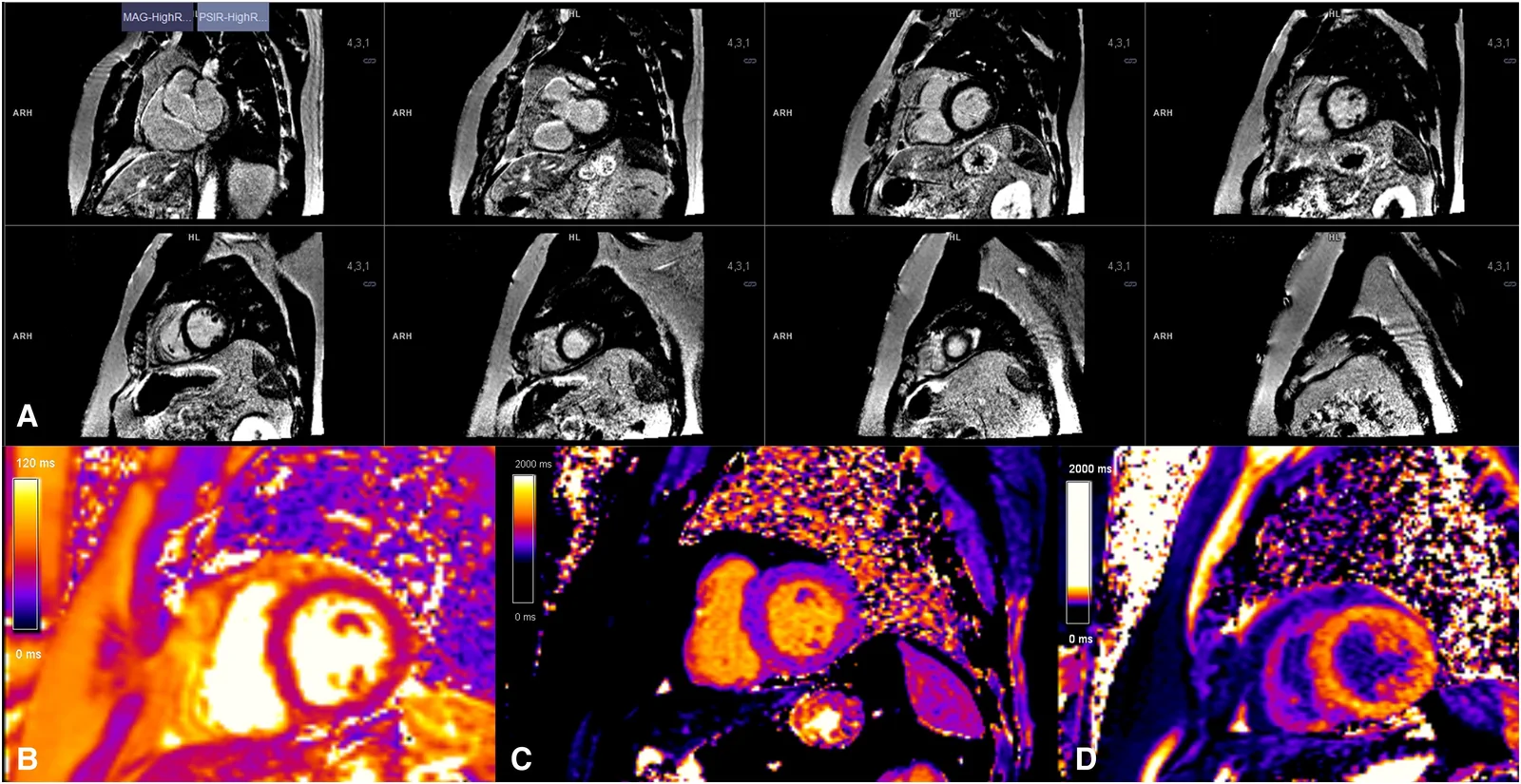
Cardiac magnetic resonance imaging demonstrating preserved myocardial tissue characteristics. (*A*) Late gadolinium enhancement imaging using a phase-sensitive inversion recovery sequence in short-axis view showing no evidence of myocardial fibrosis. (*B*) T2 myocardial mapping (short-axis, mid-ventricular level) without signs of myocardial oedema. (*C*) Native T1 myocardial mapping (short-axis, mid-ventricular level) demonstrating normal myocardial T1 values. (*D*) Post-contrast T1 myocardial mapping (short-axis, mid-ventricular level). Extracellular volume values were within the normal range, indicating the absence of diffuse myocardial fibrosis.

**Figure 4:**
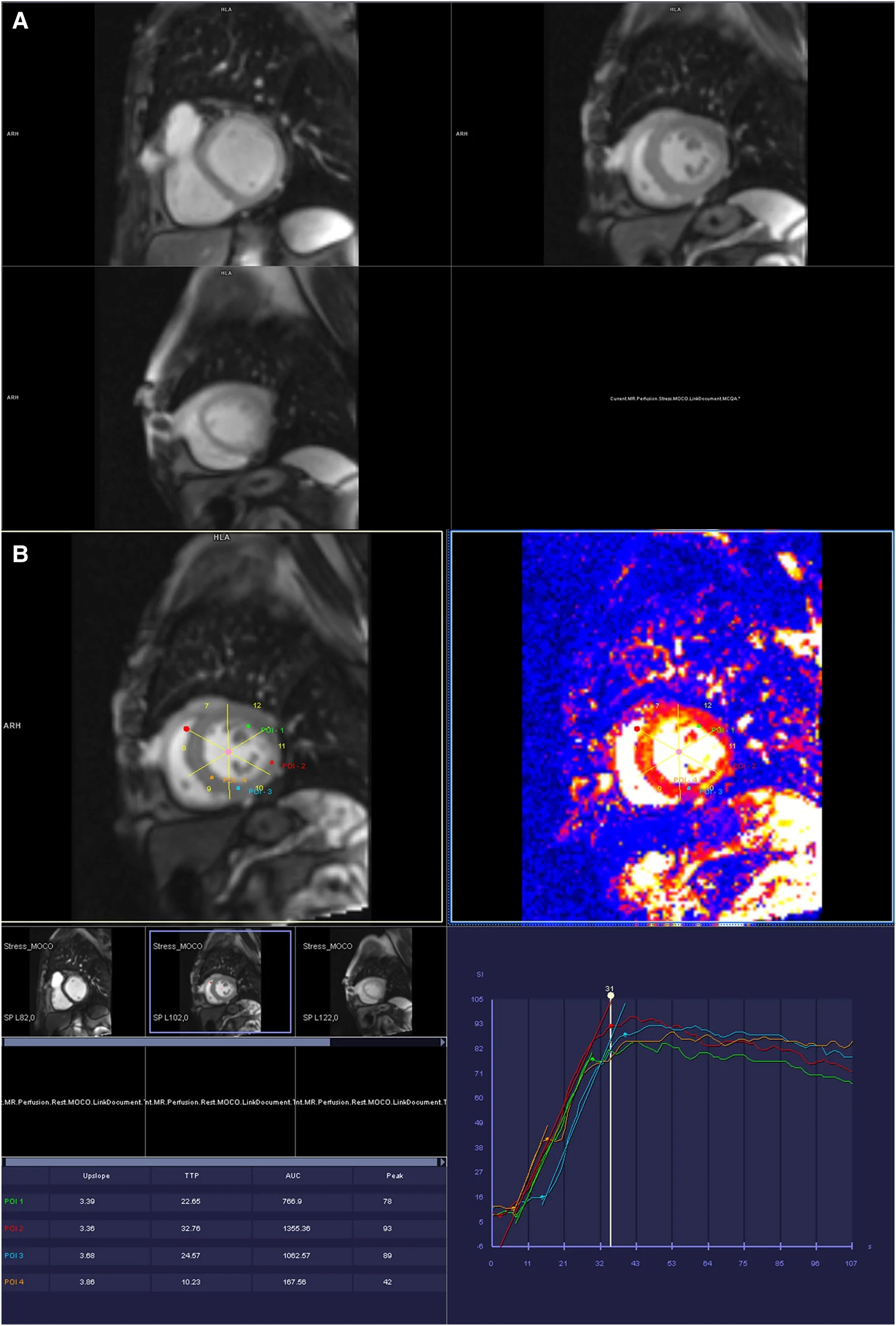
Adenosine stress perfusion cardiac magnetic resonance imaging. (*A*) Qualitative assessment of stress perfusion imaging in short-axis view showing homogeneous myocardial enhancement without regional perfusion defects. (*B*) Semi-quantitative perfusion analysis demonstrating normal myocardial perfusion curves without evidence of inducible ischaemia.

Further evaluation with multislice CT coronary angiography revealed a complex fistulous network originating from the proximal segment of the circumflex artery. The fistula followed a retroaortic course with one branch draining into the superior vena cava and another branch extending cranially in an inter-pulmoatrial course, draining into the proximal segment of the descending aorta. Additionally, a separate fistula originating from the proximal right coronary artery drained into the superior vena cava and shared a common inlet with a fistulous connection between the circumflex artery and the right atrium (*Figure 2*). The complex course and drainage of the CAFs are further illustrated in Supplementary material online, *Video S1*. Cardiac magnetic resonance (CMR) imaging demonstrated no evidence of myocardial oedema or fibrosis on T1 and T2 mapping or late gadolinium enhancement sequences. Extracellular volume values were within normal limits, and myocardial perfusion imaging on stress CMR showed no perfusion defects (*Figures 3 and 4*). Left ventricular volumes and systolic function were within normal limits, with no evidence of volume overload or haemodynamic significance of the fistulous communication (Qp:Qs = 1.07).

After a comprehensive multimodality imaging assessment, the Heart Team opted for conservative management with optimal medical therapy. The patient was discharged on rivaroxaban, beta-blockers, angiotensin-converting enzyme inhibitors, and trimetazidine. At 2-year follow-up, the patient remained clinically stable without adverse cardiovascular events.

## Discussion

We report an exceptionally rare case of multiple CAFs originating from both coronary arteries with complex and atypical drainage sites. The prevalence of CAF detected during invasive coronary angiography is ∼0.1%–0.2%, while higher detection rates have been reported with CT due to improved spatial resolution and broader use of non-invasive imaging.^1,2^ The most commonly described subtype is coronary-to-pulmonary artery fistula.^3^ Additional reports have described complex CAFs with unusual anatomical courses and drainage patterns, highlighting the marked heterogeneity of this condition. However, bilateral CAFs with multiple drainage sites remain exceptionally uncommon. Recent case reports further emphasize the importance of multimodality imaging for accurate anatomical characterization and individualized management planning.^3,4^ There is no consensus regarding the optimal management of CAFs, particularly in patients without clear evidence of myocardial ischaemia or significant haemodynamic burden. Treatment strategies are generally guided by fistula size, anatomical complexity, symptoms, and functional significance. Although surgical ligation remains an established option, late complications such as myocardial infarction have been reported.^5^ Percutaneous closure may be considered in selected cases, while conservative management with antithrombotic therapy is often appropriate in asymptomatic or mildly symptomatic patients.^3^

This case adds to the literature by demonstrating an exceptionally rare combination of bilateral CAFs with multiple atypical drainage sites, including the superior vena cava, right atrium, and descending aorta. Although similar complex fistulas have been reported, cases combining bilateral coronary origin, multiple drainage pathways, and comprehensive functional assessment with stress CMR remain extremely uncommon. In our case, multimodality imaging enabled precise anatomical and functional characterization and supported a conservative Heart Team–guided management strategy.^6,7^

Coronary artery fistulas may arise from the right coronary artery, the left coronary system, or less commonly from both coronary arteries and may drain into cardiac chambers, the pulmonary artery, vena cava, coronary sinus, or other great vessels.^8,9^ Clinical significance depends on fistula size, shunt burden, myocardial steal, and associated chamber volume overload. Management ranges from conservative follow-up in asymptomatic or haemodynamically insignificant fistulas to transcatheter or surgical closure in selected cases.^10,11^

In the absence of guideline-directed recommendations, management decisions should be individualized and made by a multidisciplinary Heart Team, integrating clinical presentation with detailed anatomical and functional imaging findings.

## Lead author biography


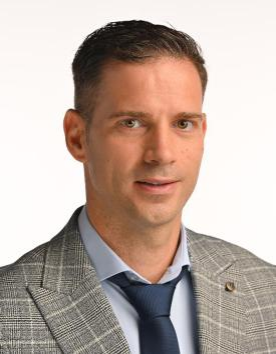


Dušan Ružičić, MD, is an interventional cardiologist at the Department of Invasive Cardiology, Health Center Valjevo, Serbia, and a faculty member at the Faculty of Health Sciences, Singidunum University. His clinical and academic interests include coronary artery disease, coronary anomalies, multimodality cardiovascular imaging, and decision-making in complex coronary interventions. He is actively involved in clinical practice, medical education, and case-based research, with a particular focus on integrating invasive and non-invasive imaging to optimize patient management.

## Supplementary Material

ytag683_Supplementary_Data

## Data Availability

The data underlying this article are available within the article and its online supplementary material.
